# New Appliances and Things Medical

**Published:** 1904-04-09

**Authors:** 


### NEW APPLIANCES AND THINGS MEDICAL.

### CHLOROS.

(The United Alkali Company, Limited, 30 James Street, Liverpool.)

This preparation has now been in use for a sufficiently long time to fully establish its position as one of the most valuable disinfectants on the market. Extended experience has verified the special advantages originally claimed for it. Briefly stated, it may be said of chloros that, while being a powerful antiseptic, it is free from unpleasant odour, it does not stain articles immersed in it, while last but not least is its exceeding cheapness, a point of much importance in these days of ever-increasing hospital expenditure. As chloros costs only 1s 9d. per gallon, and as it is an efficient germicide when diluted to the extent of one volume in 100 volumes of water, it follows that 100 gallons of disinfectant can be obtained for the small sum of 1s. 9d. This is a fact which should commend itself to the attention of all prudent hospital managers. For dealing with infected linen, etc., cleaning the floors of out-patient rooms, corridors, operating theatres, and other departments, and in various disinfecting processes where large quantities of chemical are required, chloros is an ideal disinfectant.

